# Compatibility Tests between Three Commercially Available Organic PCMs and Metals Typically Used in Fin-and-Tube Heat Exchangers

**DOI:** 10.3390/ma14185172

**Published:** 2021-09-09

**Authors:** Paulina Rolka, Jaroslaw Karwacki, Maciej Jaworski

**Affiliations:** 1 Institute of Fluid Flow Machinery, Polish Academy of Sciences, 80-231 Gdansk, Poland; jaroslaw.karwacki@imp.gda.pl; 2 Institute of Heat Engineering, Warsaw University of Technology, 00-665 Warsaw, Poland; maciej.jaworski@pw.edu.pl

**Keywords:** phase change material (PCM), metal corrosion, copper, aluminum, material compatibility, fin-and-tube heat exchanger

## Abstract

Energy storage is one of the most effective ways to increase energy savings and efficiency of heating and air conditioning systems. Phase change materials (PCMs) are increasingly used in latent heat thermal energy storage (LHTES) systems to increase their capacity. In such systems, costs are a very important factor of viability so the typical heat transfer elements like fin-and-tube heat exchangers are used to construct the LHTES. The problem of this approach is a possibility of corrosion of metals in contact with PCM that shortens the life cycle of LHTES. Therefore, the main objective of this work is an experimental study of the compatibility of metals typically used in fin-and-tube heat exchangers (copper and aluminum) with three commercially available organic PCMs (RT15, RT18HC, and RT22HC). Compatibility of PCMs with copper and aluminum was tested for a period of approximately two months, during which a total of 35 heating and cooling cycles were carried out, each with a complete phase transition of the tested materials. In the course of the tests it was assessed whether the PCM caused corrosion of the tested metals. The evaluation was based on the gravimetric method, calculation of corrosion rate, and visual observations and measurements of the features on the metal sample’s surface using optical microscope. It was determined that RT15, RT18 HC, and RT22 HC show low corrosion rates for aluminum and copper samples. The visual tests indicate that there was no change in the PCM solutions during the tests, only a sediment was observed for the samples with the combination of copper and aluminum. Microscopic examination of the surface of the samples did not show any significant surface changes, except for the aluminum samples, on the surface of which local microdefects were observed. It follows from the present results that copper and aluminum can be used to design the heat transfer surface in contact with the chosen PCMs.

## 1. Introduction

Energy consumption in the construction sector is 40% of the global value and is responsible for 36% of greenhouse gas emissions to the atmosphere [1]. Only in Europe, according to the European Union (EU) commission, half of the total energy consumption is used to heat and cool residential and nonresidential buildings, while about 84% of this energy is still produced from fossil fuels [2]. In order to reduce the consumption of fossil fuels and to reduce the warming effect, the use of technologies generating energy from renewable energy sources and energy storage systems is increasing. In the last two decades, heat and/or cold storage using latent heat thermal energy stores (LHTES) has been particularly popular.

The results of the study presented and discussed in this paper are part of research that is focused on a real industrial case where there was a mismatch between capacities of the heating system and the adsorption chiller operating in an office building’s AC system. In this situation, the mass flow rate of available hot water was too low to properly supply the adsorption chiller during peak load periods. Therefore, a thermal energy storage system with a phase change material (PCM) was suggested to reduce peak cooling load demand. The designed LHTES will be used in the chiller recoiling system and will operate in the temperature range from 14 °C to 24 °C. Therefore, PCMs whose phase change occurs in the working temperature range of the chiller recoiling system were selected for LHTES, namely RT15, RT 18 HC, and RT 22 HC. Their phase change temperatures are the ranges of 10–17 °C, 17–19 °C, and 20–23 °C, respectively. Taking into account the investment costs, it was proposed to use typical elements used in the production of fin-and-tube heat exchangers. The method of PCM containment in LHTES is important as the material is in direct contact with metal piping, plates, and housing units which can be damaged if the material is corrosive. The effectiveness and durability (service life) of LHTES mainly depends on the following factors: proper selection of PCM, storage design, and material compatibility between PCM and storage structural (construction) materials.

Material compatibility between PCM and storage structural (construction) materials could be a problem in the design and use of LHTES in some PCMs types. The interaction of PCMs with materials forming LHTES (metals and capsules inside LHTES) may result in: plastic deformation and swelling of the capsule material [3,4], absorption of PCMs by the capsule material [3,5], and corrosion of metals [3,4,6,7,8,9,10,11,12,13]. The phenomenon of corrosion is of a complex nature and more details on its engineering and theoretical aspects can be found in monographs [14,15]. There are different metal corrosion mechanisms, such as localized pitting, galvanic corrosion, erosion, fretting, de-alloying and hydrogen embrittlement, and oxidation [16]. In energy storage systems PCMs can behave as electrolytes while the storage container materials will act as anodes and corrode [16]. Common metal corrosion types caused by PCMs’ action are: metal oxidation (the PCM damages the porous surface layer of the container, which leads to its uniform perforation), pitting corrosion (corrosion starts at a point and then leads to the formation of pits in the surface of the tank or container; pitting arises when metal–metal coupling occurs in a electrolyte, which facilitates segregation of the container wall), and stress corrosion (corrosion occurs in the stress area) [6,16]. The occurrence of corrosion due to oxidation of the metal is typical of mild steel. In contrast, pitting corrosion is typical for stainless steel and aluminum, and stress corrosion is typical for stainless steel [6].

Bantová et al. [6,17] tested the compatibility of organic (Linpar17 and Linpar1820) and inorganic PCMs (SP22 and SP25) with brass, copper, aluminum, and carbon steel. On the basis of visual evaluation, corrosion rate (*CR*), and weight loss, they concluded that all metals showed good compatibility with organic PCMs. However, inorganic PCMs were discouraged for use in storage made of copper or carbon steel because these materials showed signs of surface corrosion and the color of the solution changed at the end of the experiment. Furthermore, higher *CR* values and more pronounced weight loss was found for copper and carbon steel compared to the other metals tested in inorganic PCMs. On the other hand, studies by Browne et al. [7] with organic PCMs (caprylic acid, palmitic acid, lauric acid, and Micronal) and with inorganic SP22 indicate that it is not recommended to use SP22 in storage made of aluminum due to high corrosion rates (equal to 41.45 mg/cm^2^year), or to use any of the studied PCMs in storage made of mild steel due to high mass loss (from 0.08 g up to 1.09 g) and high corrosion rate (from 2.75 mg/cm^2^year to 37.58 mg/cm^2^year). Browne et al. [7] showed that the tested PCM have the best compatibility with stainless steel and plastic Perspex. LHTES made of stainless steel is also recommended by Cabeza et al. [8] for zinc nitrate hexahydrate, sodium hydrogen phosphate dodecahydrate, and calcium chloride hexahydrate (inorganic PCMs). Moreover, sodium hydrogen phosphate dodecahydrate and calcium chloride hexahydrate are also compatible with brass. Cabeza et al. [8] also recommend placing calcium chloride hexahydrate in LHTES made of copper and suggest careful use of these PCMs with steel and aluminum (only for short-term applications) because they show a high corrosion rate (in the range of 0.2 mg/cm^2^year to 99.6 mg/cm^2^year). Cabeza et al. [9] also tested the compatibility of other PCMs: TH29 and mixtures of TH29 with MgCl_2_·6H_2_O (in a 2:1 mass ratio) with brass, copper, aluminum, steel, and stainless steel. Their test results confirmed that these PCMs are not compatible with aluminum, steel, and stainless steel. In turn, they recommend the use of TH29 and a mixture of TH29 with MgCl_2_·6H_2_O with brass and copper. Experimental tests done by Ferrer et al. [10,11] focused on the effect of organic PCMs (PureTemp23, a mixture of capric acid and myristic acid, and a mixture of capric acid and palmitic acid) as well as one inorganic PCM (SP21) on two types of stainless steel, carbon steel, copper, and aluminum. The results showed that PureTemp23 does not corrode any of these metals, but in the case of a mixture of acids, Ferrer et al. do not recommend using them in storage made of copper. Although these PCMs caused moderate corrosion rates (in the range of slightly more than 0 mg/cm^2^year to approximately 11 mg/cm^2^year), the copper corroded in a way that manifested as a blue coloration of the test tubes. The SP21 should be used carefully in storage made of aluminum or carbon steel because these metals showed signs of corrosion. Marín et al. [18] checked the material compatibility of PCMs with a phase change temperature in the temperature range of 20–25 °C for use in building installation. They tested the material compatibility of two organic PCMs (RT21 and RT25) and two inorganic (SP21E and HS-24P) with copper, aluminum, and stainless steel samples. As a result of the research, it was found that stainless steel was the most corrosion-resistant metal, with its corrosion rate ranging from 0.3 to 9.9 mg/cm^2^year, so it is recommended for long-term use. In the case of copper and aluminum samples under the influence of organic PCM, no major traces of corrosion were found, and the corrosion rate ranged from 0.3 to 9.9 mg/cm^2^year. Therefore, for RT21 and RT25, aluminum and copper can be used for long-term use. However, in contact with a PCM of inorganic origin, aluminum shows strong corrosion with corrosion rates ranging from 100 to 999 mg/cm^2^year and with visible pitting and bubbles appearing on the sample surface. Hence, the use of aluminum with SP21E and HS-24P is not recommended. Like aluminum, copper has also been corroded by the action of an inorganic PCM. Due to the observation of the characteristic blue deposit on the surface of the samples and the corrosion rate ranging from 10 to 49 mg/cm^2^year, caution is recommended when using copper as a long-term container with SP21E and HS-24P. Moreover, in order to avoid or delay the corrosion of copper with noncorroding PCMs (SP21E and HS-24P), it is suggested to cover the copper with a protective coating. Devanuri et al. [19] also investigated the material compatibility of copper, aluminum, and stainless steel with six PCMs: paraffin wax, sodium acetate trihydrate, lauric acid, myristic acid, palmitic acid, and stearic acid. These tests were carried out at two constant temperatures of 30 °C and 80 °C. During the tests, no discoloration of the solution was observed for paraffin and sodium acetate trihydrate, while for the remaining PCMs a change of solution to orange-yellow was observed for aluminum and stainless steel samples, and to green for copper samples. Due to the high corrosion rates and surface corrosion, the combination of copper with lauric, palmitic, and stearic acids should be avoided. In addition, the use of aluminum with myristic and lauric acids is not advisable as it causes pitting corrosion. Kahwaji et al. [4] noticed that fatty acids are not compatible with copper alloys, magnesium alloys, silicone rubber, and polypropylene, and sometimes it is possible to use them with nickel alloys and polyvinyl chloride (PVC). However, they are compatible with polycarbonate, stainless steel, and aluminum. On the other hand, the tests of Moreno et al. [12] showed that PCMs such as S10 or S46, C10, ZnCl_2_·3H_2_O, NaOH·1.5H_2_O, K_2_HPO_4_·6H_2_O, MgSO_4_·7H_2_O, Zn(NO_3_)_2_·4H_2_O, or K_3_PO_4_·7H_2_O are all compatible with stainless steel. Moreover, C10 and MgSO_4_·7H_2_O are also compatible with aluminum, ZnCl_2_·3H_2_O with copper, and K_3_PO_4_·7H_2_O with carbon steel. Studies conducted by Farrell et al. [13] include the impact of inorganic PCMs (PlusICE E17 and ClimSel C18) on copper, aluminum, and the samples made of a combination of copper and aluminum taken from the heat exchanger. They presented that the samples of aluminum had local pitting corrosion and that the samples which were a combination of aluminum and copper had galvanic corrosion. In the tests reported by Lazaro et al. [3] compatibility of organic PCMs (RT20, RT25, and RT27) and a nonlimiting PCM (DC 24) with plastic materials such as high-density polyethylene (HDPE), low-density polyethylene (LDPE), and polypropylene (PP) has been checked. The results of the measurements of Lazaro et al. [3] indicated that HDPE was the best material showing the least deformation. In turn, LDPE and PP showed consumption and absorption of the PCM.

Past compatibility studies between PCMs and construction materials of energy storage provide recommendations on which types of PCMs can be applied and which materials should be used in the design of LHTES (see Table 1). However, the results of these compatibility tests do not clearly present how a certain type of PCM affects a given type of material. The effect of PCM on metals and plastics varies and depends on the specific material/chemical compound. Therefore, this article presents a compatibility study between three commercial organic PCMs, with low phase transition temperatures, and construction materials of a thermal energy store. To our best knowledge, the effects of RT15, RT18 HC, and RT22 HC on samples made of copper, aluminum, and a combination of these two metals are being investigated for the first time. The experimental investigations carried out in this work show the corrosion rate of the samples, and also the effect of the PCM on their surface structure. The study of material compatibility of a PCM with a copper–aluminum combination sample has not been considered in the literature so far. The results presented in this paper highlight that it is also important to conduct PCM compatibility studies for samples with two metals in contact because they may show different effects than when each of the metals is tested separately. This aspect of the research is important for LHTES based on heat exchangers in which combinations of two metals, copper and aluminum, are common. The presented experimental results of material compatibility tests for RT15, RT18 HC, and RT22 HC provide knowledge on whether these PCMs cause corrosion in such heat exchangers.

## 2. Materials and Methods

### 2.1. Materials

**Figure 1:**
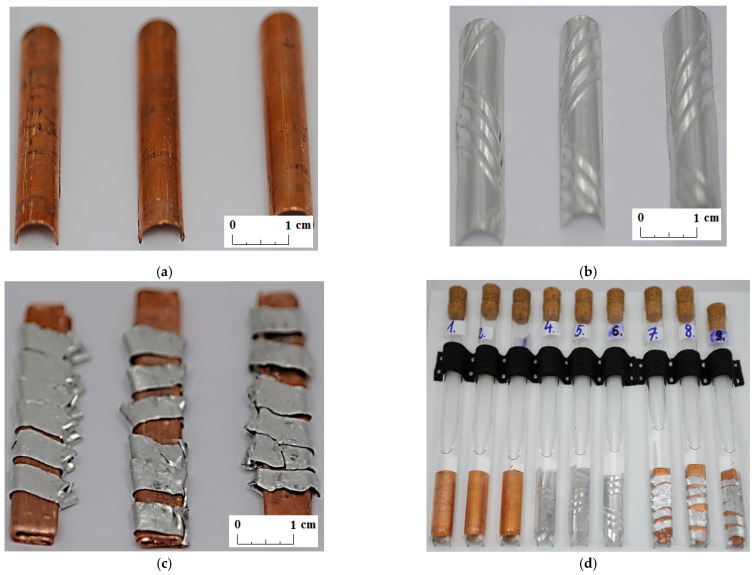
Tests samples: (**a**) copper; (**b**) aluminum; (**c**) combination of copper and aluminum; and (**d**) a view of all test samples placed in PCM tubes.

In the experiment, it was decided to determine the compatibility between commercially available organic PCMs and materials found in a typical fin-and-tube heat exchanger, which could be used as the structural material of the LHTES. Copper samples, aluminum samples, and samples with a combination of copper and aluminum were prepared for testing (see Figure 1). The metal samples were cut from the heat exchanger. The dimensions (width × length) of the prepared metal samples were as follows: aluminum sample 2.4 cm× 4.1 cm, copper sample 1.9 cm × 3.7 cm, and copper–aluminum combination sample 1.0 cm × 4.1 cm.

In this study three commercial PCMs, RT15, RT18 HC, and RT22 HC, were investigated. These PCMs can be used to support heating or cooling systems in buildings and have low phase transition temperatures that fall within the range of 10 °C to 23 °C [20,21,22,23,24]. PCM samples of volume amounting to 10 mL were applied to glass tubes with a diameter of 16 mm and a length of 160 mm (except tube No. 9 which had a diameter of 16 mm and a length of 150 mm). Then metal samples were put into test tubes. Table 2 shows the distribution of metal samples in the tubes with PCM.

### 2.2. Methods

Compatibility studies between organic PCM and metal samples were carried out using two measurement methods, the gravimetric method and the visual method.

Compatibility assessment by gravimetric method is based on the determination of mass loss and corrosion rate (*CR*) due to placing metal samples in contact with a PCM over a specified period of time.

The gravimetric method (experiment) consists of the following steps:

1. Preparation of the sample: samples are cut out, grinded with sandpaper, and cleaned (usually with acetone);
2. Weighing of samples: metal samples are weighed before being put into the test tubes filled with the PCM;
3. Immersion of samples in the PCM: metal samples are placed into tubes filled with the PCM (at a temperature when the PCM is a liquid) and they are completely immersed in the PCM, after which they are kept at a constant temperature (when the PCM is a liquid) [7,8,9,10,11] or are tested in temperature cycles triggering phase transformation processes [3,4,5,6,13,17];
4. Removal of samples from the PCM: metal samples are removed from the PCM after a specified time (e.g., a week, a month, or several months);
5. Sample cleaning-up: metal samples are cleaned and dried after being removed from the PCM;
6. Reweighing of samples: metal samples are weighed and subjected to visual evaluation;
7. Corrosion rate calculation: based on the measurement results, the mass change of the sample and the corrosion rate (*CR*) are determined.

Corrosion rate (mg/cm^2^/year), taking into account the weight loss (Δ*m*), the surface area of the metal/metal alloy sample (*A*), and the experiment time (*t_o_* − *t*), is calculated based on the relationship [4,6,7,8,9,10,11,12,17]:

(1) $$CR=\frac{\Delta m}{A\;\cdot \;\left(t_{o}-t\right)}$$

where

(2) $$\Delta m=m\left(t_{o}\right)-m\left(t\right)$$

The calculated corrosion rates are compared to the corrosion weight loss guide used in the industry according to [28]. By referring to the aforementioned guidelines, it is possible to determine whether the PCM is compatible with the sample material or not. Namely, if the corrosion rate is less than 10 mg/cm^2^year it is recommended to use the metal in LHTES construction for long term service. If the corrosion rate is in the range from 10 to 49 mg/cm^2^year, caution is recommended as material compatibility depends on the specific application. Finally, if the corrosion rate is greater than 50 mg/cm^2^year, the materials are not compatible and their use is not recommended [28].

In the paper [13] the authors also present the determination of the corrosion rate based on ASTM G1 standard [29], where on the basis of short-term (several hours) tests it is possible to determine the average corrosion rate. It is defined by the following equation [13,29]:

(3) $$CR=\frac{K\cdot W}{A\cdot t\cdot D}$$

where *K*—A constant (equal 1 × 10^4^·D [g/m^2^h]), *W*—Mass loss [g], *t*—Time of exposure in hours [h], and *D*—Density [g/cm^3^].

In turn, the visual method is based on the microscopic measurement of the features at the surface of the metal samples and on the basis of visual observations during tests, such as change in color of the solution, separation of sediment, or bubble formation.

### 2.3. Experimental Setup

**Figure 2:**
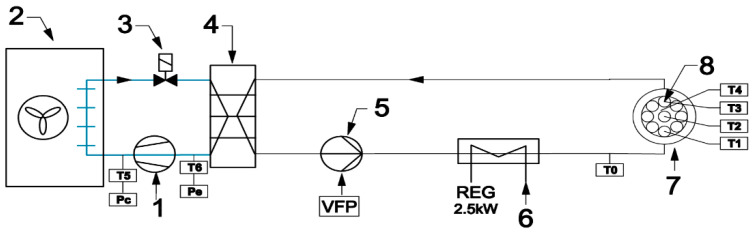
Layout of experimental setup: (**1**) compressor; (**2**) condenser; (**3**) electronic expansion valve; (**4**) evaporator; (**5**) circulating pump; (**6**) heater; (**7**) water bath; and (**8**) samples.

An experimental setup was designed and built to test samples during repeated melting and solidification cycles. Its layout is shown in Figure 2. Two individual loops separated by the heat exchanger (4) can be distinguished. The first loop (left of Figure 2) is a typical compressor chiller. A commonly used thermostatic expansion valve was replaced by an electronic device (3) to attain a more stable operation. An algorithm of valve control reduced the fluctuations of the working parameters. In the second loop, water circulated as the heat transfer fluid. To ensure a fast temperature response of the system, the amount of water was minimized. The samples (8) are placed in a water bath (7). Water chilled in the evaporator (4) was pumped to the circuit immersed in the water bath (7) by the variable speed circulating pump (5). A heat transfer fluid flow rate was kept constant by controlling the pump rotating speed. The flow rate in all investigated cases was set to 120 kg/h. The electric heater (6) maintained the set inlet temperature of the water. To minimize the fluctuations of this temperature, the heater was equipped with the precise single-phase power controller JUMO TYA-201 (JUMO GmbH, Fulda, Germany). The applied control systems allowed for high precision of experimental conditions.

**Figure 3:**
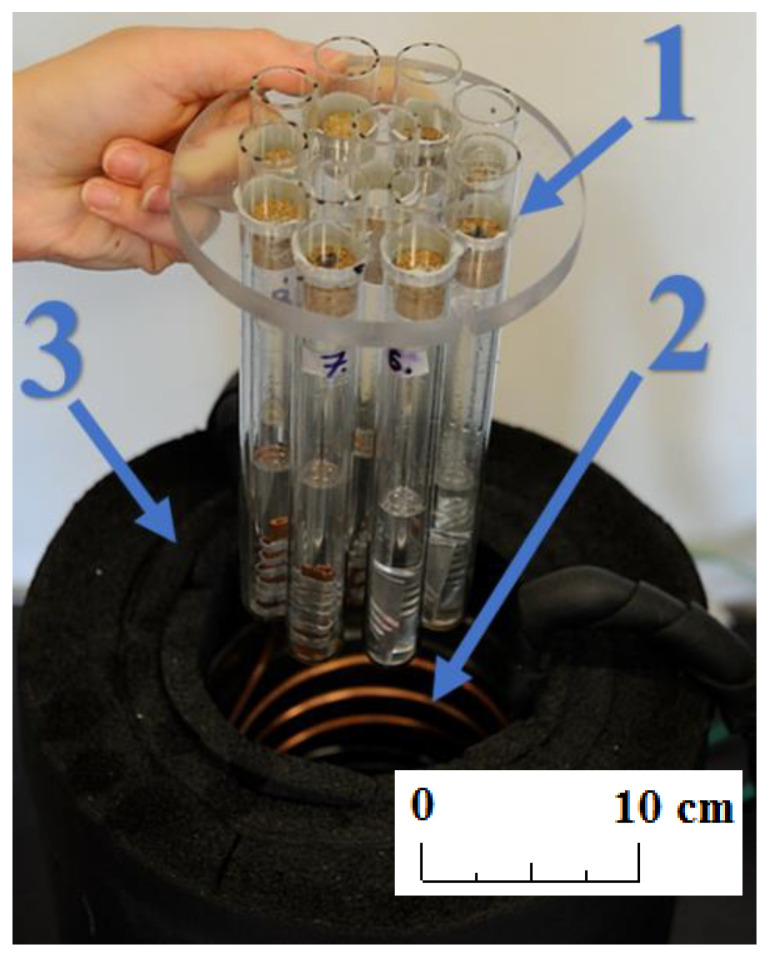
Photo of the experimental stand; (**1**) samples; (**2**) water bath and heat exchanger; and (**3**) insulation.

Four K-type thermocouple sensors with 1 mm shield diameter were installed in the center of four samples containing different PCMs. Sensor T0, located upstream of the water bath, was used to control the electric heater. All thermocouples were calibrated and tested before installation using the Beamex MC6 calibrator (BEAMEX OY AB, Pietarsaari, Finland), the DRUCK DB-150 (GE-Sensing, Billerica, MA, USA) calibration furnace, and the reference temperature sensor PT100 ISOTECH (Isothermal Technology Ltd., Southport, England). After that, the precision of the thermocouples was ±0.1 °C for 0 °C and ±0.15 °C for 30 °C. The value of the standard deviation of the temperature measurement was 0.15 K. Pressure transducers (marked as Pe and Pc in Figure 2) were used by the refrigerant cycle safety and control system. A view of the experimental setup is shown in Figure 3.

**Figure 4:**
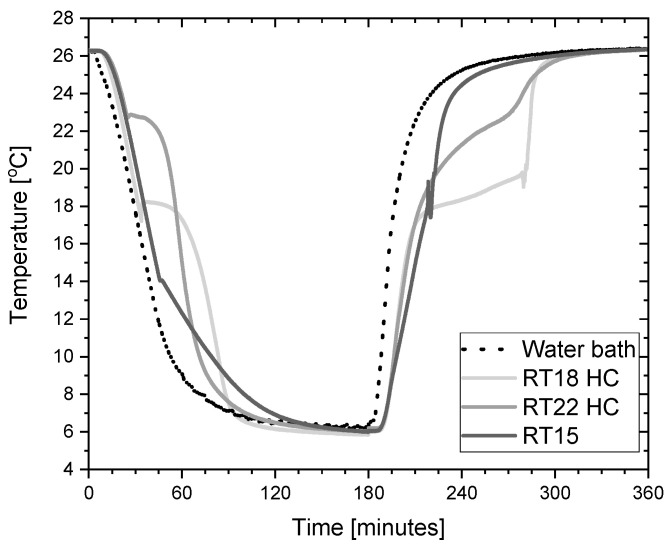
Typical temperature trends of the PCM and water bath during a typical cooling and heating cycle in testing of these materials.

The presented experimental stand allowed us to conduct tests in an automatic mode, consisting of the cyclic increase and decrease of the temperature. The system was switched between the temperatures of 4 °C and 26 °C. An example of the cooling and heating cycle of the PCM during testing is shown in Figure 4.

## 3. Results and Discussion

Compatibility between the PCM (RT15, RT18 HC, and RT22 HC) and metal samples (copper, aluminum, and a copper/aluminum combination) was checked for a period of 7 weeks. This time span is similar to compatibility investigations by Marín et al. [18] and Devanuri et al. [19]. Metal samples were extracted from the PCM after 1, 3, 4, and 7 weeks for weighing. A precision laboratory balance RADWAG PS 8000/C/1 (RADWAG, Radom, Poland) was used to measure the weight of the metal samples. The accuracy of the balance reading is 10 mg, its repeatability is 15 mg, and linearity equals to ±30 mg [30]. The results of the mass measurements and the calculated corrosion rates are shown in Table 3 and Table 4.

Based on the results in Table 3, no significant changes in the weight of the samples made of copper and aluminum were found. The value of the corrosion rate coefficient for samples made of the pressed elements of the fin-and-tube heat exchanger is negative. In this case there was likely a problem with removing the PCM from the space between the aluminum and the copper without destroying the sample. This caused an increase in the mass of the sample during the measurements resulting in a negative value of the *CR* coefficient. It can be concluded that the gravimetric method is not suitable for samples with complex geometry and the values of *CR* for the sample numbers 7 to 9 are unreliable. For this reason, the visual assessment of samples and the PCM is of primary importance.

**Figure 5:**
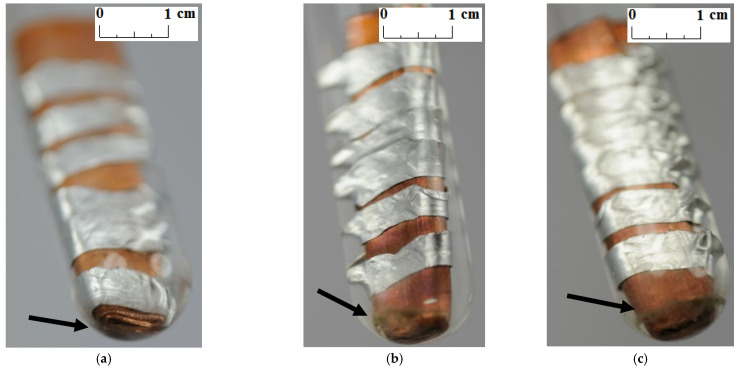
The precipitate which deposited on the sample with a combination of aluminum and copper immersed in RT15; (**a**) after 3 weeks; (**b**) after 4 weeks; and (**c**) after 7 weeks.

**Figure 6:**
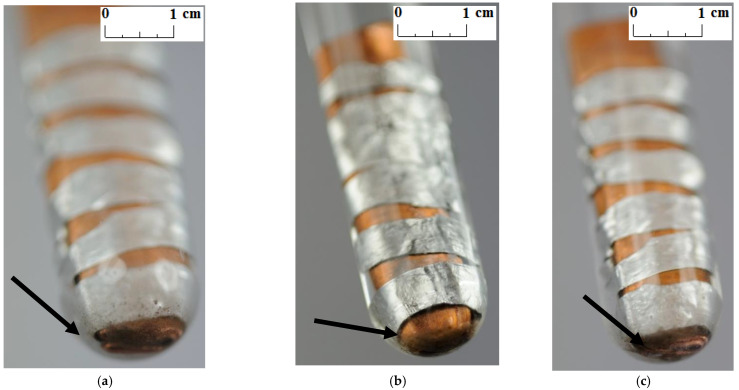
The precipitate which deposited on the sample with a combination of aluminum and copper immersed in RT18 HC; (**a**) after 3 weeks; (**b**) after 4 weeks; and (**c**) after 7 weeks.

**Figure 7:**
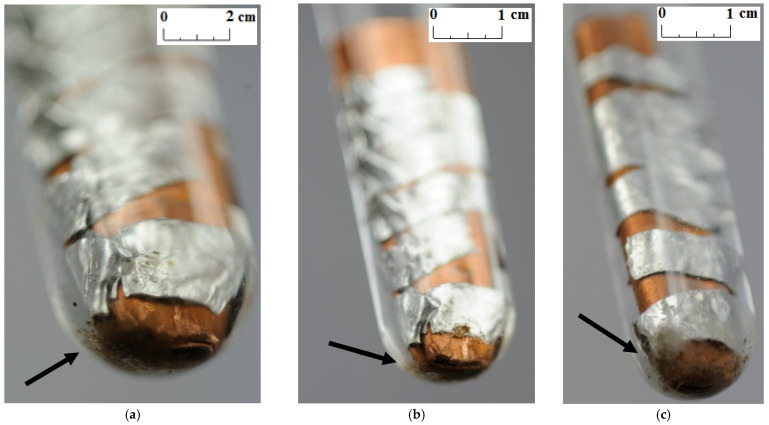
The precipitate which deposited on the sample with a combination of aluminum and copper immersed in RT22 HC; (**a**) after 3 weeks; (**b**) after 4 weeks; and (**c**) after 7 weeks.

Unlike other studies [4,7,11,12], no change in the color and transparency of the PCMs was observed in any of the samples during the course of the test. Moreover, no gas bubbles were noticed on the metal surface. On the other hand, a precipitate was observed on the bottom of the test tubes in which the samples made of a combination of copper and aluminum were placed (see the arrows in Figure 5, Figure 6 and Figure 7). It was visible since the third week of testing. The amount of precipitate seems to have increased slightly over time. However, it is difficult to determine whether the sediment on the bottom of the tubes was caused by interaction between the PCMs and metal samples. These are not significant changes; however, it is advisable to conduct research over a longer period of time.

**Figure 8:**
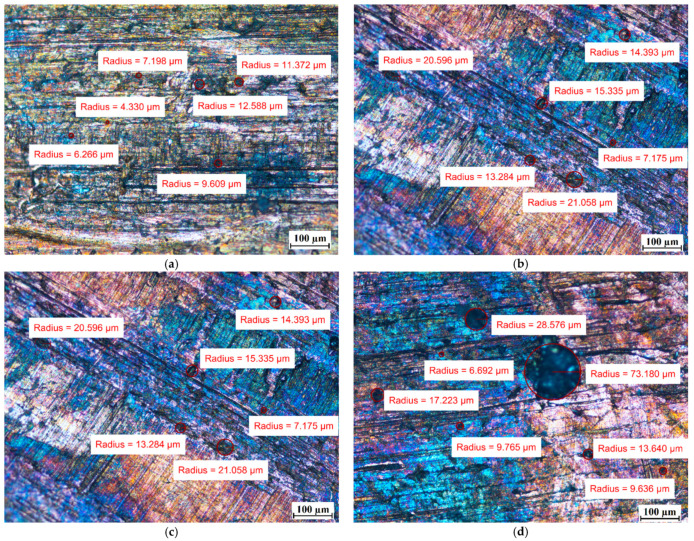
The surface of the copper samples (Cu) (**a**) before immersion in PCM; (**b**) after 7 weeks immersion in RT15; (**c**) after 7 weeks immersion in RT18 HC; and (**d**) after 7 weeks immersion in RT22 HC.

**Figure 9:**
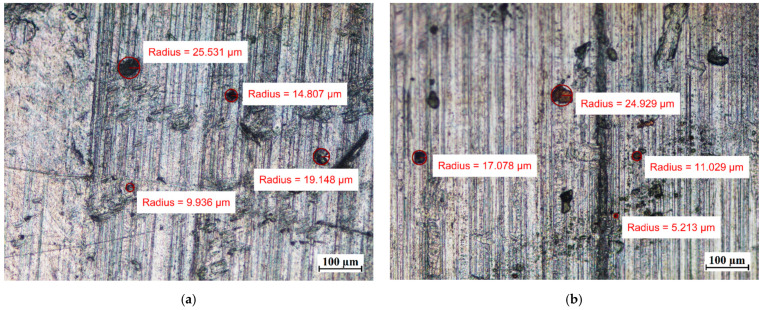

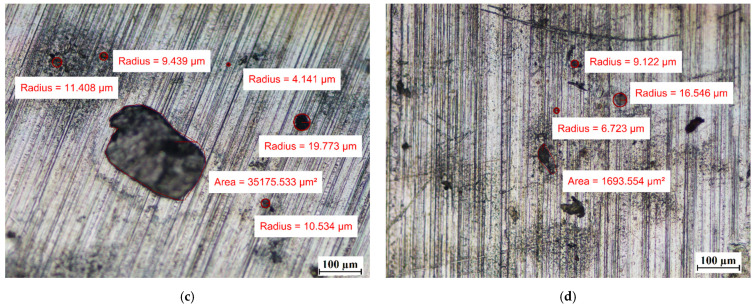
The surface of the aluminum samples (Al) (**a**) before immersion in PCM; (**b**) after 7 weeks immersion in RT15; (**c**) after 7 weeks immersion in RT18 HC; and (**d**) after 7 weeks immersion in RT22 HC.

**Figure 10:**
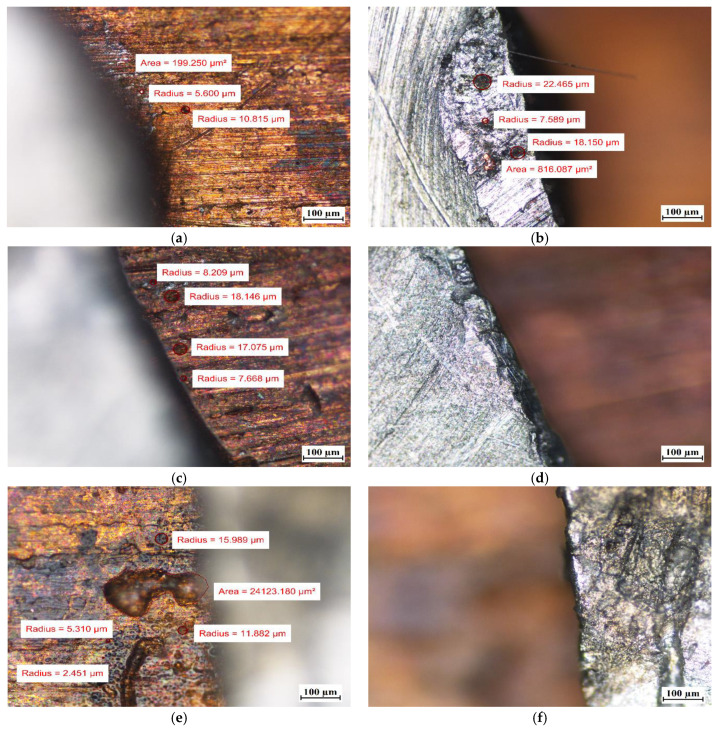

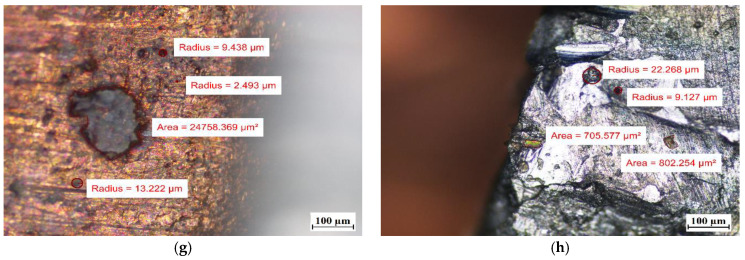
The surface of samples with a combination of copper and aluminum (Cu-Al) (**a**) before immersion in PCM–copper side; (**b**) before immersion in PCM–aluminum side; (**c**) after 7 weeks immersion in RT15–cooper side; (**d**) after 7 weeks immersion in RT15–aluminum side; (**e**) after 7 weeks immersion in RT18 HC–copper side; (**f**) after 7 weeks immersion in RT18 HC–aluminum side; (**g**) after 7 weeks immersion in RT22 HC–copper side; and (**h**) after 7 weeks immersion in RT22 HC–aluminum side.

Visual tests were also performed to evaluate the effect of the PCM on the surface of the metal samples. The surface features of the metal samples were measured using NIKON ECLIPSE Ti-S optical microscope (NIKON INSTRUMENTS Inc., Melville, NY, USA) with a Nikon DS-Fi2 camera (NIKON INSTRUMENTS Inc., Melville, NY, USA). Metal surface examinations using optical microscopy were performed before the tests and after 7 weeks of immersion of the metal samples in the PCM. The results of the visual measurement (with ten times magnification) are shown in Figure 8, Figure 9 and Figure 10.

In particular, attention was paid to the presence of discoloration on the surface of the samples and changes in their numbers and sizes during the tests.

For the copper samples, the presence of rather large areas of discoloration on their surface was noted even before they were immersed into the PCM. Discoloration of copper samples was also reported in [13] during material compatibility tests with PCMs ClimSel C18 and E17, where it was found that the discoloration was caused by chemical corrosion due to chloride ion concentration on the surfaces. However, in our research, it is difficult to determine whether chemical corrosion has occurred because the exact chemical composition of the PCMs tested is unknown.

Comparing the surface of the samples that had no contact with PCMs with those that were immersed in PCMs, the degradation of the surface of aluminum was noticed. Although the surface of the aluminum samples showed defects with diameters ranging from ca. 10 µm to ca. 25 µm before immersion in the PCM, after 7 weeks of immersion the degradation increased. In addition to the areas with defects of diameters ranging between ca. 4 µm and 25 µm, the formation of local areas with numerous microdefects also showed up. Based on [13,31], it was assumed that the visible damage was due to corrosion processes, namely the pitting microcorrosion. The presence of pitting microcorrosion was visible for the aluminum samples placed in RT18 HC and RT22 HC.

Therefore, long-term studies are recommended to ascertain the further effect of these PCMs on aluminum. Based on microscopic observations and measurements of the copper samples, it can be concluded that no significant changes on their surface were noticed.

## 4. Conclusions

In the industrial installation, there was a problem with powering the chiller from the heating network. In order to cover the peak demand, it was proposed to use a latent heat thermal energy storage (LHTES) with fin-and-tube heat exchangers, which would not generate too much investment cost. This approach made it necessary to check whether there would be any corrosion of the LHTES materials in contact with the PCM.

An experimental compatibility test between three commercially available organic PCMs (RT15, RT18 HC, and RT22 HC) and two metals (coper and aluminum) was conducted. In order to assess whether there was corrosion of copper and aluminum as a result of contact with RT15, RT18 HC, and RT22 HC, a gravimetric method was used (with calculations of the corrosion rate) together with visual observations of the changes inside the test tubes containing the metal and PCM samples. The visual inspection and gravimetric analysis were made during two months of the experiments. Based on the results from the gravimetric measurements it can be concluded that no significant changes in the mass of the samples made only of copper and only of aluminum were found. For the samples with a combination of copper and aluminum, it was difficult to measure the mass variation over time. Although great care has been taken to clean the samples thoroughly, most likely some residual PCM remained in the sample gaps that resulted in an unreliable increase in the samples’ masses in the course of the experiment. For these samples, eventual corrosion presence was determined by visual methods only. Closer inspection of the samples’ surfaces using an optical microscope revealed that the copper samples were not deteriorated by the action of a PCM to an extent that would have practical significance. Only enlargement of areas of discoloration on the surface was observed. On the other hand, in the case of the aluminum samples, the formation of local areas of surface microdefects, which look like pitting microcorrosion, was noticed. Moreover, during the tests of the samples with a combination of copper and aluminum, since the third week of testing, a formation of sediment on the bottom of the test tubes was observed. The amount of precipitate seems to have increased slightly over time. However, it is difficult to determine whether the sediment on the bottom of the test tubes was caused by interaction of the PCMs with the metal samples.

Summing up the results of the presented experimental research, it can be concluded that the fin-and-tube heat exchanger made of copper and aluminum can be recommended for the construction of LHTES using organic PCMs RT15, RT18 HC, and RT22 HC. However, due to the local presence of microdefects on the aluminum samples, caution should be exercised. Long-term studies are required to quantitatively identify the long-term effects of RT15, RT18 HC, and RT22 HC on aluminum and a copper–aluminum combination. Therefore, the authors of the study plan to continue the research for samples of these two types in the future.

## Figures and Tables

**Table 1:** List of recommendations regarding compatibility between PCM and potential LHTES materials or encapsulation materials.

**PCM**	**Type of PCM**	**Recommendation**											**Ref.**
		**Carbon Steel**	**Stainless Steel**	**Mild Steel**	**Steel**	**Aluminum**		**Copper**	**Copper and Aluminum**	**Brass**	**Plastic/** **Perspex**	**Nylon**	
Linpar 17	Organic	R	NI	NI	NI	R		R	NI	R	NI	NI	[6,17]
Linpar 1820	Organic	R	NI	NI	NI	R		R	NI	R	NI	NI	[6,17]
SP21 (Rubitherm)	Inorganic	CR	R	NI	NI	NR ^1^		R	NI	NI	NI	NI	[10,11]
SP22 (Rubitherm)	Inorganic	CR ^1^	R	R ^1^	NI	NR ^1^		R ^1^	NI	R	NI	R	[6,7,17]
SP25 (Rubitherm)	Inorganic	CR ^1^	NI	NI	NI	R		R ^1^	NI	R ^1^	NI	NI	[6,17]
PureTemp 23 (PureTemp)	Organic	R	NI	R	NI	R		R	NI	NI	NI	NI	[10,11]
Caprylic acid and palmitic acid	Organic	R	R	NI	NI	R		CR ^1^	NI	NI	NI	NI	[10,11]
Caprylic acid and myristic acid	Organic	R	R	NI	NI	R		NR ^1^	NI	NI	NI	NI	[10,11]
Caprylic acid	Organic	NI	R	CR ^1^	NI	CR ^1^		CR ^1^	NI	CR ^1^	R	NI	[7]
Palmitic acid	Organic	NI	R	CR ^1^	NI	CR ^1^		CR ^1^/NR	NI	CR ^1^	R	NI	[7,19]
Lauric acid	Organic	NI	R	CR ^1^	NI	CR ^1^		CR ^1^	NI	CR ^1^	R	NI	[7,19]
Myristic acid	Organic	NI	R	NI	NI	NR ^1^		CR ^1^	NI	NI	NI	NI	[19]
Stearic acid	Organic	NI	R	NI	NI	CR ^1^		NR ^1^	NI	NI	NI	NI	[19]
Micronal (BASF)	Organic	NI	R	CR ^1^	NI	R		R	NI	R	R	NI	[7]
Zn(NO_3_)_2_∙6H_2_O	Inorganic	NI	R	NI	NR ^1^	NR ^1^		NR ^1^	NI	NR	NI	NI	[8]
Na_2_HPO_4_·12H_2_O	Inorganic	NI	R	NI	CR ^1^	NR		CR ^1^	NI	R	NI	NI	[8]
CaCl_2_·6H_2_O	Inorganic	NI	R	NI	CR ^1^	CR ^1^		R ^1^	NI	R	NI	NI	[8]
TH29 (TEAP)	Inorganic	NI	CR ^1^	NI	NR ^1^	NR ^1^		R	NI	R	NI	NI	[9]
mix TH29 and MgCl_2_·6H_2_O	Inorganic	NI	CR ^1^	NI	NR ^1^	NR^1^		R	NI	R	NI	NI	[9]
C10	Organic	NI	R	NI	NI	R		NR ^1^	NI	NI	NI	CR ^1^	[4]
C12	Organic	NI	R	NI	NI	R		CR ^1^	NI	NI	NI	CR ^1^	[4]
C14	Organic	NI	R	NI	NI	R		CR ^1^	NI	NI	NI	CR ^1^	[4]
C16	Organic	NI	R	NI	NI	R		CR ^1^	NI	NI	NI	CR ^1^	[4]
C18	Organic	NI	R	NI	NI	R		NR ^1^	NI	NI	NI	CR ^1^	[4]
Octadecanol	Organic	NI	R	NI	NI	R		R	NI	NI	NI	CR^1^	[4]
PlusICE E17 (PCM Products)	Inorganic	NI	NI	NI	NI	NR ^1^		R	NR^1^	NI	NI	NI	[13]
ClimSel C18 (ClimSel)	Inorganic	NI	NI	NI	NI	NR ^1^		R	NR^1^	NI	NI	NI	[13]
RT21 (Rubitherm)	Organic	NI	R	NI	NI	R		R	NI	NI	NI	NI	[18]
RT25 (Rubitherm)	Organic	NI	R	NI	NI	R		R	NI	NI	NI	NI	[18]
SP21E (Rubitherm)	Inorganic	NI	R	NI	NI	NR ^1^		CR ^1^	NI	NI	NI	NI	[18]
HS-24P (Rgees)	Inorganic	NI	R	NI	NI	NR ^1^		CR ^1^	NI	NI	NI	NI	[18]
Paraffin wax	Organic	NI	R	NI	NI	R		R	NI	NI	NI	NI	[19]
Sodium acetate trihydrate	Organic	NI	R	NI	NI	R		R	NI	NI	NI	NI	[19]
**PCM**	**Type of PCM**	**Recommendation**											**Ref.**
		**Alloy of Magnesium (AZ91D)**	**Alloy of Nickel and** **Silver (C7521)**	**HDPE**	**LDPE**	**PET**	**PP**	**Acrylic**	**Poly-** **carbonate**	**PCV**	**Silicone** **Rubber**	**ABS**	
C10	Organic	NR ^1^	NR ^1^	NI	NI	NI	NR ^1^	NR ^1^	R	NR ^1^	NR ^1^	NR ^1^	[4]
C12	Organic	CR ^1^	NR ^1^	NI	NI	NI	NR ^1^	NR ^1^	R	CR ^1^	NR ^1^	NR ^1^	[4]
C14	Organic	CR ^1^	NR ^1^	NI	NI	NI	NR ^1^	NR ^1^	R	CR ^1^	NR ^1^	NR ^1^	[4]
C16	Organic	R	NR ^1^	NI	NI	NI	NR ^1^	CR ^1^	R	CR^1^	NR ^1^	NR ^1^	[4]
C18	Organic	NR ^1^	NR ^1^	NI	NI	NI	NR ^1^	CR ^1^	R	R	NR ^1^	CR ^1^	[4]
Octadecanol	Organic	R	R	NI	NI	NI	NR ^1^	CR ^1^	R	R	NR ^1^	CR ^1^	[4]
TH24 (Teap)	Inorganic	NI	NI	R	NR	R	NI	NI	NI	NI	NI	NI	[3]
DC24 (Cosella Dörken)	Inorganic	NI	NI	R	NR	NI	NI	NI	NI	NI	NI	NI	[5]
RT20 (Rubitherm)	Organic	NI	NI	NI	NR	R	CR	NI	NI	NI	NI	NI	[3,5]
RT25 (Rubitherm)	Organic	NI	NI	NI	NR	R	CR	NI	NI	NI	NI	NI	[3,5]
RT26 (Rubitherm)	Organic	NI	NI	NI	NR	R	CR	NI	NI	NI	NI	NI	[3,5]
RT27 (Rubitherm)	Organic	NI	NI	NI	NR	R	CR	NI	NI	NI	NI	NI	[3,5]

^1^ corrosion effect; R—Recommended; CR—Caution recommended; NR—Not recommended; NI—No information.

**Table 2:** Distribution of metal samples in tubes with PCM.

Sample Number	1	2	3	4	5	6	7	8	9
Sample material	Cu	Cu	Cu	Al	Al	Al	Cu-Al	Cu-Al	Cu-Al
PCM	RT15	RT18 HC	RT22 HC	RT15	RT18 HC	RT22 HC	RT15	RT18 HC	RT22 HC
PCM melting temp [°C]	10–17	17–19	20–23	10–17	17–19	20–23	10–17	17–19	20–23
PCM congealing temp [°C]	17–10	19–17	23–20	17–10	19–17	23–20	17–10	19–17	23–20
Heat storage capacity [kJ/kg]	155	260	190	155	260	190	155	260	190
Reference	[25]	[26]	[27]	[25]	[26]	[27]	[25]	[26]	[27]

**Table 3:** The results of the mass measurements.

Sample Number	Mass of Metal Sample [g]				
	Before Experiment	After 1 Week	After 3 Weeks	After 4 Weeks	After 7 Weeks
1	3.67 ± 0.03	3.67 ± 0.03	3.67 ± 0.03	3.66 ± 0.03	3.66 ± 0.03
2	3.72 ± 0.03	3.72 ± 0.03	3.72 ± 0.03	3.70 ± 0.03	3.72 ± 0.03
3	3.35 ± 0.03	3.35 ± 0.03	3.35 ± 0.03	3.32 ± 0.03	3.35 ± 0.03
4	0.72 ± 0.03	0.71 ± 0.03	0.71 ± 0.03	0.70 ± 0.03	0.71 ± 0.03
5	0.65 ± 0.03	0.65 ± 0.03	0.65 ± 0.03	0.65 ± 0.03	0.65 ± 0.03
6	0.57 ± 0.03	0.58 ± 0.03	0.59 ± 0.03	0.58 ± 0.03	0.61 ± 0.03
7	7.58 ± 0.03	7.64 ± 0.03	7.73 ± 0.03	7.68 ± 0.03	7.75 ± 0.03
8	7.97 ± 0.03	8.00 ± 0.03	8.09 ± 0.03	8.10 ± 0.03	8.07 ± 0.03
9	7.43 ± 0.03	7.46 ± 0.03	7.55 ± 0.03	7.58 ± 0.03	7.55 ± 0.03

**Table 4:** Calculated corrosion rates.

Sample Number	CR [mg/cm^2^year]			
	After 1 Week	After 3 Weeks	After 4 Weeks	After 7 Weeks
1	0	0	0	0
2	0	0	0	0
3	0	0	0	0
4	0	0	0	0
5	0	0	0	0
6	0	0	0	0
7	−763	−636	−318	−309
8	−382	−509	−413	−182
9	−382	−509	−477	−218

## Data Availability

Data is contained within the article.

## Nomenclature

*A*	area
*CR*	corrosion rate
*D*	density
Δ*m*	the weight loss
*K*	constant
*m*	mass
*t*	time
*W*	mass loss
